# Should Be Removed or Taken Biopsy in Early Stage: A Retrospective Study of 953 Cases of Nevus Sebaceous From a Single Center

**DOI:** 10.1111/1346-8138.17941

**Published:** 2025-09-10

**Authors:** Wange Zhou, Yu Cao, Chenglong Wu, Kaili Zhou, Xue Zhang, Dan Deng

**Affiliations:** ^1^ Department of Dermatology Xinhua Hospital, Shanghai Jiaotong University School of Medicine Shanghai China; ^2^ Institute of Dermatology Shanghai Jiaotong University School of Medicine Shanghai China; ^3^ Institute of Dermatology, Shanghai Children Medical Center Shanghai Jiaotong University School of Medicine Shanghai China

**Keywords:** BCC, benign tumors, nevus sebaceous, optimal timing of prophylactic excision, secondary tumors

## Abstract

No consensus was made on whether all Nevus sebaceous (NS) should undergo prophylactic excision and the best age of surgery. This is a retrospective study. Patients who underwent surgery and were confirmed as NS by pathology during January 2014 to December 2023 in the Department of Dermatology of Xinhua hospital were included in this study. A total of 953 cases were included, composed of 536 males and 417 females. Most patients underwent their first surgery as adolescents (mean 12.5 years). Scalp (66.4%) and face (26.1%) were the most common sites. General anesthesia was rarely used in patients older than 5 years in this series. Fifty‐two tumors developed in 46 cases, including 44 benign and 8 malignant (BCC). The most common benign tumors arose from hair follicles (22 tumors) and apocrine glands (17 tumors), especially syringocystadenoma papilliferum (*n* = 14), trichilemmoma (*n* = 10), and trichoblastoma (*n* = 6). In patients aged 5–12 years, 13 benign tumors and 2 BCC cases (ages 10.1, 10.3 years) were observed, with the youngest being a 4.7‐year‐old with SCAP. We identified 15.1 years as the cut‐off age of tumor presence in the resected NS lesion. At this single center, diffuse and multifocal lesions tended to be resected at a younger age than lesions located on the face. Resection of NS led to a marked reduction in patient‐reported psychological discomfort burden scale (*p* < 0.05). The malignant tumor rate was low. However, in patients under 12 years, 13 benign tumors and 2 BCC cases were observed. Given benign tumor occurrence in childhood and BCC over 10 years, monitoring should begin at 10 years and resection is recommended before 15.1 years as tumor risk increases. Suspicious lesions should be excised regardless of age. Resection of NS could improve psychological well‐being.

## 1 Introduction

Nevus sebaceous (NS), also known as organoid nevus, is a benign hamartomatous skin lesion characterized by ectopic sebaceous gland hyperplasia. NS typically presents at birth or in early childhood, with an estimated incidence of 3 in 1000 live births. The prevalence appears similar between sexes. Occasionally, there is a positive family history [1, 2].

While largely benign, NS can significantly impact cosmesis and psychology, especially when located on the face or scalp. NS exhibits various distributions including Blaschko linear, quadrant, patchy, and checkerboard patterns. Some exhibit a cerebriform surface. Mutations early in development tend to follow Blaschko lines, while later mutations are more circumscribed [3, 4].

The natural history of NS involves three stages [5]: early infancy with papillomatous hyperplasia and immature hair follicles; pubertal expansion due to hormones, with sebaceous gland and apocrine gland maturation; and the adult stage where benign or malignant tumors may form, including but not limited to trichoblastoma (TB), syringocystadenoma papilliferum (SCAP), sebaceous epithelioma (SE), basal cell carcinoma (BCC), trichilemmoma, adnexal carcinoma, and eccrine poroma [6, 7].

Controversy remains around prophylactic excision and optimal timing [8, 9, 10, 11, 12, 13, 14, 15, 16]. While some advocate early excision, others do not advocate for early excision as older patients can undergo local anesthesia instead of general anesthesia. Others suggest excision at or before puberty onset [6, 17]. There is also limited literature describing pediatric NS. Management considerations include surgery risks versus monitoring, patient and family preferences, and malignant transformation risks over time.

We aim to provide insights on optimal NS management based on our 10‐year experience. Through retrospective analysis of patient characteristics, pathology, and outcomes, we compare our findings to previous studies to identify best practices around surgery timing and anesthesia choice.

## 2 Materials and Methods

### 2.1 Case Series Study

This study was approved by the Ethics Committee of Xinhua Hospital, Shanghai, China (No. XHEC‐D‐2021‐155). A retrospective analysis of 953 patients was performed using the Dermatologic department pathology database from January 2014 to December 2023 by searching for ‘nevus sebaceous’. For multiple procedures, only the first procedure on the same patient was included. Age at surgery ranged from 0.1 to 75.2 years, divided into infants (0–1 years), toddlers (1–5 years), children (5–12 years), adolescents (12–18 years), adults (18–50 years) and middle‐aged/elderly (> 50 years). Local anesthesia for some young children was assisted by oral chloral hydrate. Follow‐up questionnaires were completed by 118 patients collecting data on onset, location, surface characteristics, cerebriform appearance, symptoms, other system involvement, clinical diagnosis, pre‐surgical treatments, postoperative interventions, scar satisfaction ratings, and pre‐ versus postoperative psychological burden scale using a 10‐point scale (0 = no impact, 10 = extreme discomfort).

### 2.2 Literature Review and Data Extraction

See Supporting Information—S1.

### 2.3 Statistical Analysis

All statistical analyses were performed using SPSS 26 and R 4.3.1. The McNemar's test was used to detect differences in binary variables between first surgery and initial discovery. Paired samples t‐test compared pre‐ and post‐surgical psychological burden scales. Pearson and Spearman correlation analyzed associations between continuous variables, after assessing normality using Kolmogorov–Smirnov. When analyzing lesion length–age correlation, multifocal or diffuse lesions were excluded due to length measurement difficulty. The secondary tumor age cut‐off was determined using smoothed accuracy curves from a logistic regression tumor prediction model, calculating classification accuracies across age cut‐offs to optimally balance early detection and premature intervention risks.

## 3 Results

### 3.1 Clinical Findings

We conducted a retrospective review of 953 patients who underwent NS excision surgery between January 2014 and December 2023. There were 536 males and 417 females, with ages at first surgery ranging from 0.1 to 75.2 years (mean: 12.5, median: 9.7). Most patients (661, 69.4%) were under 12 years old. The largest proportion (48.4%) underwent first surgery during childhood (5–12 years) (Figure S1a). Table 1 displays the demographic and clinical data.

**TABLE 1:** Demographic and clinical data of 953 patients with nevus sebaceous.

Age (years)	
Mean ± SD	12.5 ± 12.0
Range	0.1–75.2
Age at surgery, *n* (%)	
(0,1)	52 (5.5)
(1,5)	148 (15.5)
(5,12)	461 (48.4)
(12,18)	125 (13.1)
(18,50)	146 (15.3)
(50,100)	21 (2.2)
Gender, *n* (%)	
Male	536 (56.2)
Female	417 (43.8)
Locations, *n* (%)	
Face	236 (24.8)
Head	623 (65.4)
Neck/shoulder/chest	46 (4.8)
Trunk	26 (2.7)
Limbs	6 (0.6)
Diffused	3 (0.3)
Face and head	8 (0.8)
Face and head and neck/shoulder/chest	2 (0.2)
Face and neck	3 (0.3)
Clinical diagnosis (*n*, %)	
Sebaceous nevus	643 (67.5)
Verrucous nevus	251 (26.3)
Skin mass	40 (4.2)
Warts	6 (0.6)
Sebaceous gland hyperplasia	3 (0.3)
Seborrheic keratosis	2 (0.2)
Xanthoma	4 (0.4)
Congenital skin dysplasia	1 (0.1)
Sebaceous adenoma	1 (0.1)
Superficial lipomatous nevus	1 (0.1)
Papilloma	1 (0.1)

*Note:* Percentages in brackets indicate the proportion of 953 patients with NS.

Abbrevations: NS, Nevus sebaceous; SD, Standard deviation.

A total of 402 patients had outpatient surgery, while 551 were inpatients (Table 2). The highest proportion of inpatients was aged 0–5 years, with a relative downward trend from 5 to 50 years. General anesthesia was mainly used in infants/toddlers and rarely for ages 5–12 years (Table 2). Only one over 12 requested general anesthesia due to fear of pain.

**TABLE 2:** Clinical history of NS patients in different age groups.

Clinical	0–1 year (*n* = 52)	1–5 years (*n* = 148)	5–12 years (*n* = 461)	12–18 years (*n* = 125)	18–50 years (*n* = 146)	> 50 years (*n* = 21)	Total (*N* = 953)
Hospitalization, *n* (%)	42 (80.8)	122 (82.4)	283 (61.4)	50 (40.0)	44 (30.1)	10 (47.6)	551 (57.8)
General anesthesia, *n* (%)	25 (48.1)	60 (40.5)	11 (2.4)	0 (0)	1 (0.7)	0 (0)	97 (10.2)
Pruritus, *n* (%)	6 (26.1)	39 (39.8)	76 (31.8)	11 (29.7)	21 (56.8)	8 (80.0)	161 (36.3)
Rough surface, *n* (%)	21 (65.6)	52 (61.2)	226 (88.3)	47 (97.9)	41 (100)	8 (100)	395 (84.0)
Hyperplasia surface, *n* (%)	12 (48.0)	22 (30.6)	177 (80.5)	40 (100)	32 (100)	7 (100)	290 (73.0)

*Note:* The percentages in brackets indicate the proportion of patients within each specified age group.

Abbrevations: NS, Nevus sebaceous.

**FIGURE 1:**
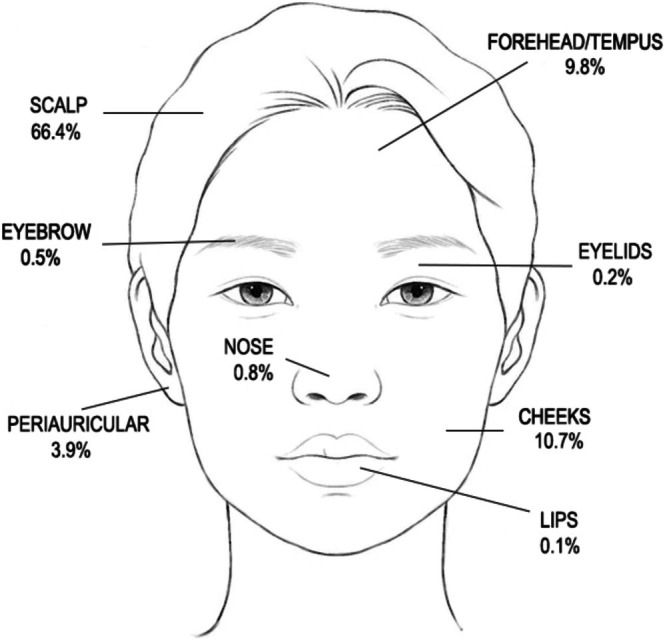
Proportional distribution of sebaceous nevi on the head and facial areas.

**FIGURE 2:**
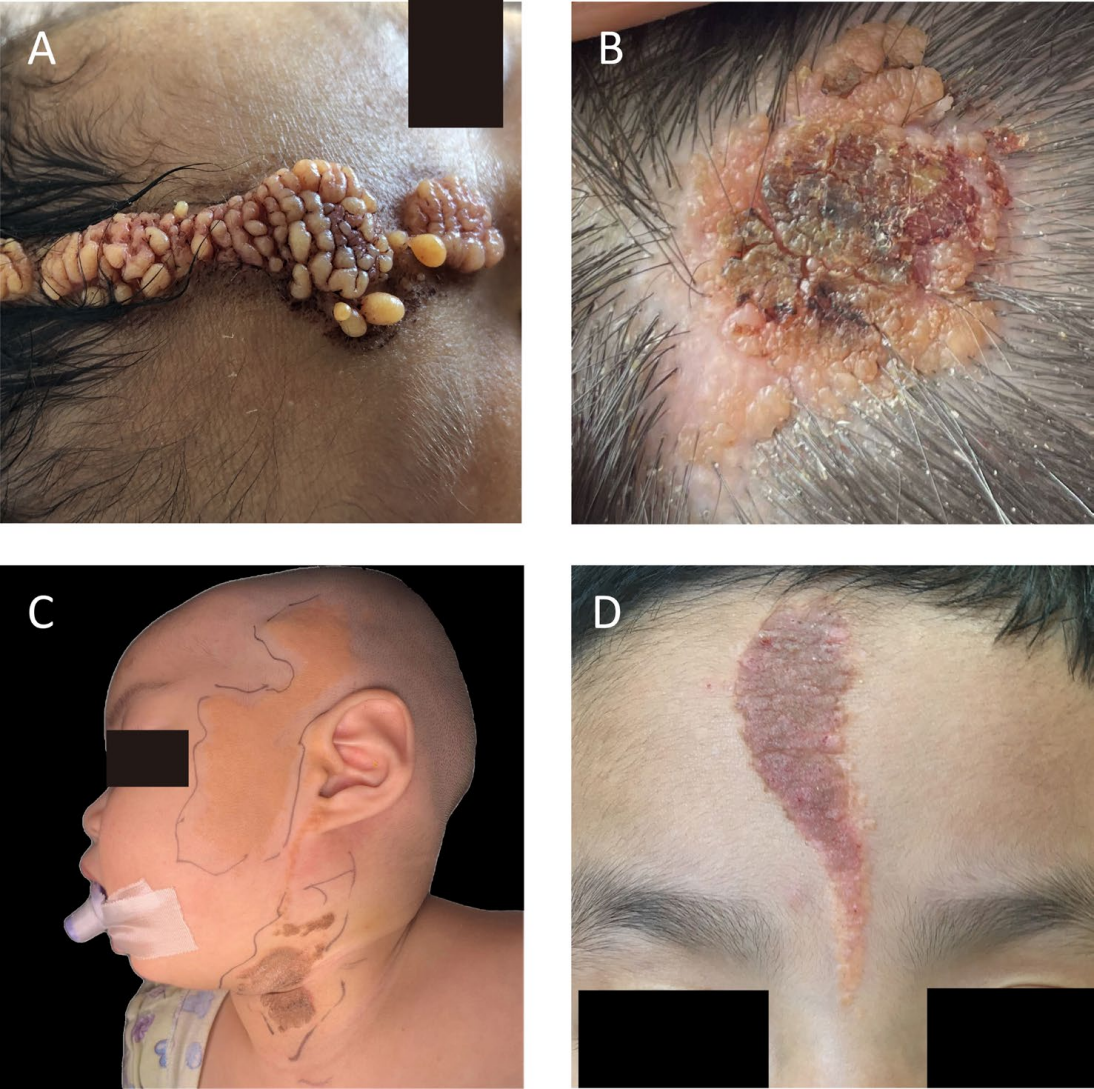
Typical clinical images of NS lesions that are aesthetically concerning or symptomatic. (A) A case of NS with gyriform appearance. (B) A case of NS accompanied with eczema. (C) A case of NS located on the face, head, and neck that severely affects aesthetic appearance. (D) A case of NS located on the forehead that severely affects aesthetic appearance.

Most lesions were on the scalp (66.4%), followed by the face (26.1%) which are aesthetically sensitive areas. Figure 1 shows the prevalence and distribution of NS across the head and face, with the cheeks being most common, followed by the forehead, temporal region, and periauricular area. Figure 2C,D show two cases of facial NS which affect aesthetic appearance, causing psychological burden. Non‐facial/scalp lesions were a small minority (Table 1). At this single center, patients with multifocal/diffuse lesions tended to undergo excision at a younger mean age (6.7 years) than those with isolated facial lesions (13.3 years), with marginal statistical significance (0.05 < *p* < 0.1, Figure S2a). No difference in age at first surgery was found for other sites (Figure S2b).

Lesion size varied greatly in the 551 inpatient surgeries, with length from 0.5 to 20 cm (mean: 3.6 cm) and width from 0.3 to 10 cm (mean: 1.9 cm). However, lesion length showed no correlation with age at surgery (*p* > 0.05).

Prior to pathological confirmation, the most common clinical misdiagnoses (Table 1) were verrucous nevus (251 cases, 26.3%), followed by skin mass (40 cases, 4.2%), and warts (6 cases, 0.6%). The rate of overall clinical misdiagnoses was 32.5% (Table 1).

**FIGURE 3:**
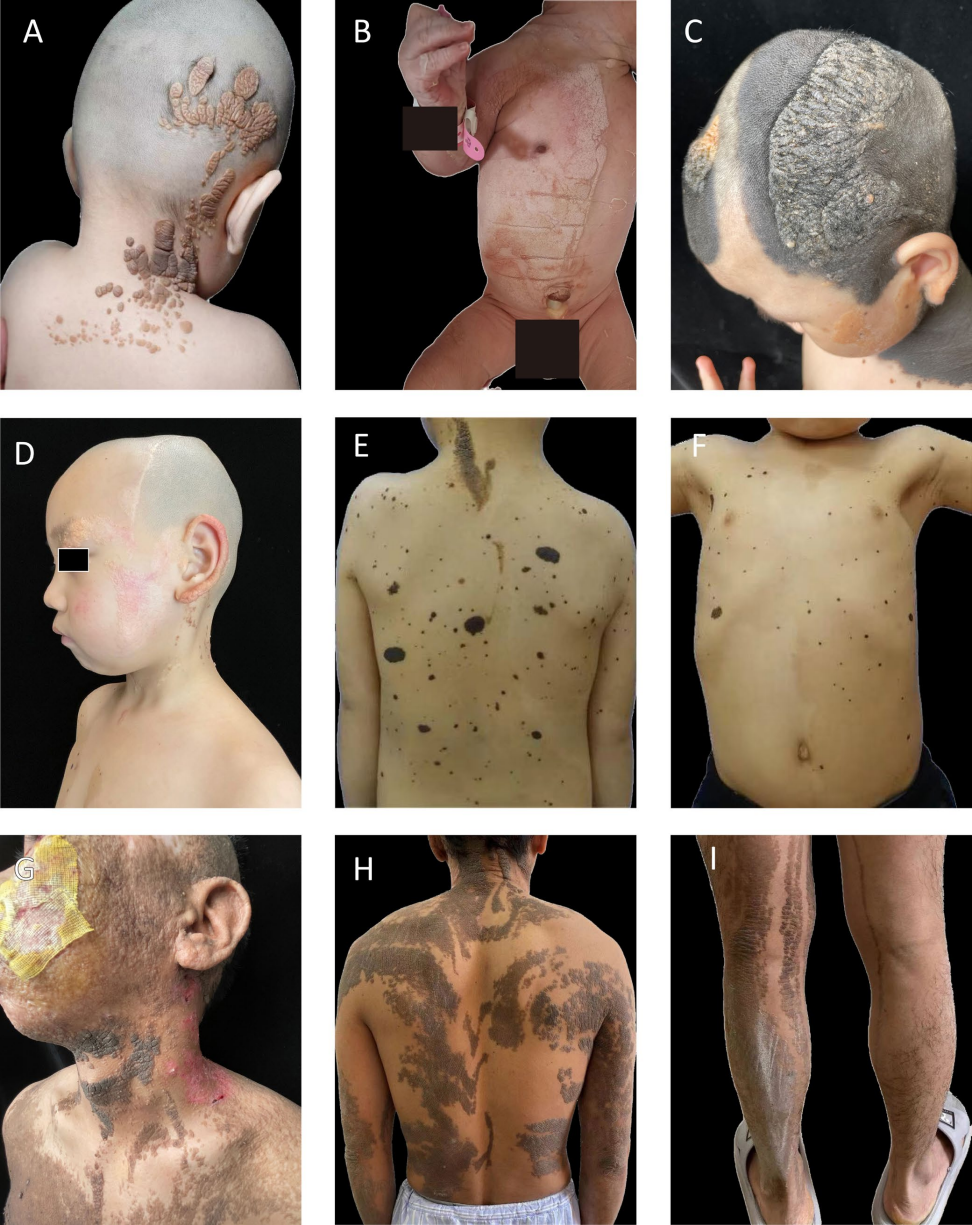
Clinical images of several typical patients with epidermal naevus syndrome. (A) Well‐demarcated diffuse brown macules and patches with varied sizes distributed along Blaschko's lines on the right scalp, right neck, and back; some had a gyriform appearance. (B) Diffused NS on the trunk, limbs, neck, and thighs. Large patches of yellow‐brown macules and papules were observed in a linear distribution on the right trunk and limbs. The lesions exhibited distinct borders with rough, slightly elevated surfaces. Some papules had a prominent pink base that was clearly elevated above the skin with a verrucous texture. (C) Large area of NS on the scalp and face, with gyriform appearance of the NS on the scalp, accompanied by Nevus giganteus. (D) (E) (F) Skin lesions of a patient with NS syndrome. Large areas of NS on the head, face, ear, and neck, accompanied by Café‐au‐lait spot and Melanocytic nevus. Yellowish‐red macules and patches on the left side of the face and neck, coffee‐brown‐black macules and patches on the trunk and limbs for 4 years. Ophthalmologic examination revealed congenital tortuosity of retinal blood vessels. Electroencephalogram showed mild abnormalities. (G) (H) (I) Skin lesions of a patient with NS syndrome. Lesions exhibited rough, irregular surfaces with some demonstrating papillomatous changes. A diffused distribution was observed over the scalp as well as predominant involvement of the left side of the face and lower extremities along Blaschko's lines.

Among the 953 NS patients, isolated NS was seen in 99.5%, while 5 (0.5%) had multisystem involvement (Figure 3) consistent with Epidermal naevus syndrome (see supplemental materials).

### 3.2 Pathological Findings

**FIGURE 4:**
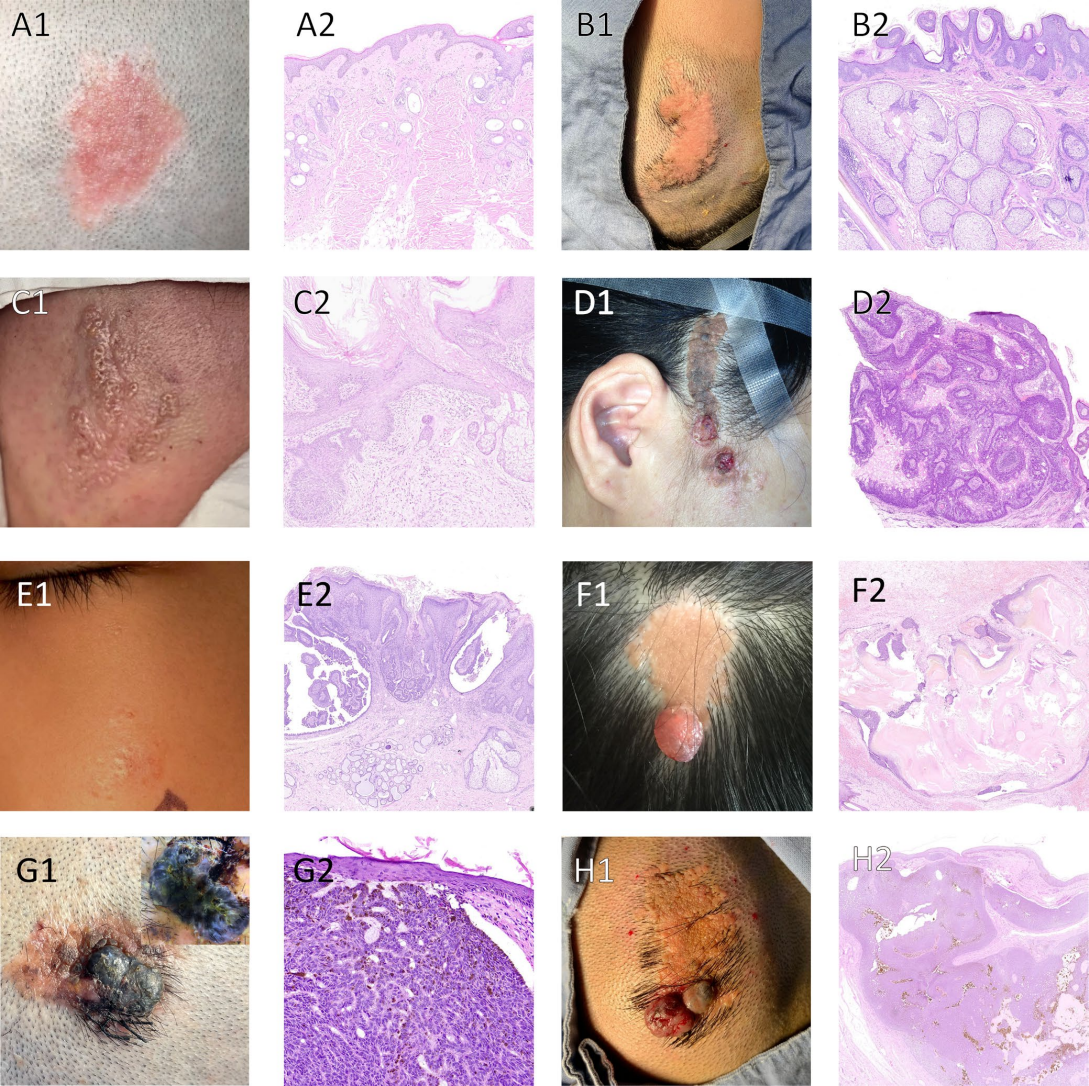
Paired clinical (A1, B1, C1, D1, E1, F1, G1, H1) and histological (A2, B2, C2, D2, E2, F2, G2, H2) images of NS at different ages; some were complicated by secondary tumors. (A1) A 5‐year‐old NS case with a smooth and flat surface. (A2) Pathology shows the epidermis showing no obvious hyperplasia. Immature sebaceous glands and immature hair follicles can be seen. (H&E; original magnification ×5). (B1) A 11.5‐year‐old case of NS with a rough and proliferated surface. (B2) Pathology shows the epidermis demonstrating papillomatous‐like hyperplasia. Within the dermis, there are numerous immature sebaceous glands seen. (H&E; original magnification ×5). (C1) A 54.4‐year‐old case of NS accompanied by trichoblastoma. (C2) Pathology shows the squamous epithelium demonstrating papillomatous‐like hyperplasia. Within the dermis, there are scattered mature and immature hair follicle sebaceous gland structures observed. Focally, basaloid cells show budding or strand‐like proliferation extending downward, consistent with NS accompanied by trichoblastoma. (H&E; original magnification ×10). (D1) A 50.8‐year‐old case of NS accompanied by sebaceous adenoma. (D2) Pathology shows focal basaloid cellular hyperplasia, consistent with sebaceous adenoma. (H&E; original magnification ×3). (E1) A 12.7‐year‐old case of NS accompanied by SCAP. (E2) Pathology shows SCAP demonstrating cystic invaginations extending downward from the epidermis, with sebaceous hyperplasia. (H&E; original magnification ×4). (F1) A 6.9‐year‐old case of NS accompanied by Pilomatrixoma. (F2) Pathology shows the characteristic of Pilomatrixoma. (H&E; original magnification ×3). (G1) Clinical picture and dermatoscopic findings of a 69‐year‐old case of NS accompanied by BCC. (G2) Pathology shows tumor cell nests composed of basaloid‐appearing cells within the dermis. The peripheral cells are arranged in a palisading pattern, and contraction clefts can be identified. (H&E; original magnification ×10). (H1) A 31.1‐year‐old case of NS accompanied by BCC. (H2) Pathology shows tumor cell nests composed of basophilic cells, with contraction clefts. (H&E; original magnification ×3)

Our main analyses included histological subtypes of secondary tumors, their derivation, and patient age distribution (Table 3). Figure 4 showed typical clinical and pathological features at different NS stages.

**TABLE 3:** Tumors associated with NS (*N* = 953) and tumor numbers in different age groups.

Source	Tumor	Age range (years) (mean)	M/F	Location (S/F/T)	*N*	% of tumors (excluding warts, *n* = 52)	% of total NS (*n* = 953)	Number of tumors across different age groups					
								(0,1)	(1,5)	(5,12)	(12,18)	(18,50)	(50,100)
Apocrine glands	SCAP	4.7–39.6 (19.6)	3/11	13/1/0	14	26.9%	1.5%	0	2	2	3	7	0
	Apocrine nevus	12.5	1/0	1/0/0	1	1.9%	0.1%	0	0	0	1	0	0
	Apocrine cyst adenoma	35.7	1/0	1/0/0	1	1.9%	0.1%	0	0	0	0	1	0
	Ductal sweat gland adenoma	10.8	0/1	1/0/0	1	1.9%	0.1%	0	0	1	0	0	0
Hair follicles	Trichilemmoma	5.4–75.2 (25.6)	9/1	6/2/2	10	19.2%	1.0%	0	0	5	1	2	2
	TB	9.4–73.6 (36.7)	2/4	5/1/0	6	11.5%	0.6%	0	0	2	0	2	2
	Trichoepithelioma	65.3	0/1	0/0/1	1	1.9%	0.1%	0	0	0	0	0	1
	Epidermoid cyst	27.0–35.7 (31.4)	2/0	2/0/0	2	3.8%	0.2%	0	0	0	0	2	0
	EVHCs	16.2	1/0	1/0/0	1	1.9%	0.1%	0	0	0	1	0	0
	PTC	27	1/0	0/1/0	1	1.9%	0.1%	0	0	0	0	1	0
	Pilomatrixoma	6.9	0/1	1/0/0	1	1.9%	0.1%	0	0	1	0	0	0
Sebaceous glands	Sebaceous adenoma	50.8	0/1	0/1/0	1	1.9%	0.1%	0	0	0	0	0	1
	Sebaceoma	32.2	1/0	1/0/0	1	1.9%	0.1%	0	0	0	0	1	0
Sweat glands	Solid‐cystic hidradenoma	45.9	0/1	1/0/0	1	1.9%	0.1%	0	0	0	0	1	0
	Eccrine poroma	56.1	0/1	1/0/0	1	1.9%	0.1%	0	0	0	0	0	1
Squamous epithelium	KA	32.2	1/0	1/0/0	1	1.9%	0.1%	0	0	0	0	1	0
	Viral warts	4.0–47.8 (15.4)	4/11	2/13/0	15	—	1.6%	0	1	8	3	3	0
Squamous epithelium	BCC	10.1–69.0 (37.3)	4/4	6/2/0	8	15.4%	0.8%	0	0	2	0	4	2

Abbreviations: BCC, basel cell carcinoma; EVHCs, eruptive vellus hair cysts; KA, Keratoacanthoma; PTC, proliferating trichilemmal cyst; S/F/T, scalp/face/trunk; SCAP, syringocystadenoma papilliferum; TB, Trichoblastoma; NS, Nevus sebaceous.

Among the 953 cases, 52 tumors (excluding warts) developed in 46 patients, including 44 benign and 8 malignant (BCC). Forty patients (4.2%) had benign tumors and 8 (0.8%) had BCC. In 40 cases, one tumor type developed per lesion. In 6 cases, two tumor types developed per lesion (Table 4). The most common benign tumors originated from hair follicles (22 tumors) and apocrine glands (17 tumors). Others originated from sebaceous glands (2 tumors), sweat glands (2 tumors), and squamous epithelium (1 tumor). The most frequent benign tumor was SCAP (14 cases, 1.5%), followed by trichilemmoma (10 cases, 1.0%) and trichoblastoma (6 cases, 0.6%).

**TABLE 4:** The frequency of histological features within nevus sebaceous lesions among different age groups.

	0–1 year (*n* = 52)	1–5 years (*n* = 148)	5–12 years (*n* = 461)	12–18 years (*n* = 125)	18–50 years (*n* = 146)	> 50 years (*n* = 21)
Cell atypia, *n* (%)	0 (0)	1 (0.7)	17 (3.7)	4 (3.2)	22 (15.1)	5 (23.8)
Mild atypia	0 (0)	0 (0)	9 (2.0)	2 (1.6)	6 (4.1)	1 (4.8)
Mild to moderate atypia	0 (0)	1 (0.7)	1 (0.2)	1 (0.8)	3 (2.1)	1 (4.8)
Moderate atypia	0 (0)	0 (0)	2 (0.4)	1 (0.8)	5 (3.4)	0 (0)
Moderate to severe	0 (0)	0 (0)	0 (0)	0 (0)	1 (0.7)	1 (4.8)
Severe atypia	0 (0)	0 (0)	3 (0.7)	0 (0)	6 (4.1)	2 (9.5)
Epidermal proliferation, *n* (%)	7 (13.5)	23 (15.5)	157 (34.1)	66 (52.8)	82 (56.2)	15 (71.4)
Inflammatory infiltration, *n* (%)	0 (0)	10 (6.8)	18 (3.9)	14 (11.2)	19 (13.0)	2 (9.5)
Granulomatous reaction, *n* (%)	0 (0)	1 (0.7)	3 (0.7)	1 (0.8)	1 (0.7)	0 (0)
Sebaceous hyperplasia, *n* (%)	2 (3.8)	5 (3.4)	33 (7.2)	11 (8.8)	22 (15.1)	6 (28.6)

*Note:* The percentages in brackets indicate the proportion of patients within each specified age group.

In pediatric patients between 5 and 12 years old, 13 secondary tumors excluding warts arose (Table 3), including 2 with BCC who were 10.1 and 10.3 years old, respectively. In patients younger than 5 years old, only 2 benign tumors were observed, with the youngest a 4.7‐year‐old with SCAP.

With increasing age, rates of epidermal proliferation, cell atypia, sebaceous gland hyperplasia (Table 4), and benign tumor rates (Table 5) generally increased. Figure 4A2 and 4B2 visually depict the histopathological changes in the epidermis and sebaceous glands that occur with increasing age. Figure 4 C2, 4 D2, 4 E2, and 4 F2 show pathological pictures of a few representative cases of benign neoplasms. The average age of patients with benign tumors (27.0 years) was significantly higher than that of those without (*p* < 0.001, Figure S3a), indicating the correlation of age at surgery with the presence of benign tumors.

**TABLE 5:** Tumors associated with nevus sebaceous according to age range.

Age range (years)	Benign tumors^a^	Malignant tumors	Multiple tumors	Age (pathology)^b^
(0,1)	0 (0%)	0 (0%)	0 (0%)	—
(1,5)	2 (1.4%)	0 (0%)	0 (0%)	—
(5,12)	11 (2.4%)	2 (0.4%)	1 (0.2%)	10.3 (TB + BCC)
(12,18)	6 (4.8%)	0 (0%)	1 (0.8%)	12.5 (SCAP+ Apocrine nevus)
(18,50)	18 (12.3%)	4 (2.7%)	4 (2.7%)	32.2 (Sebaceoma+KA); 35.3 (SCAP+TB); 35.7 (Apocrine cyst adenoma+Epidermoid cyst); 45.9 (Solid‐cystic hidradenoma+BCC)
(50,100)	7 (33.3%)	2 (9.5%)	0 (0%)	—

*Note:* The percentages in brackets indicate the proportion of patients within each specified age group.

Abbrevations: BCC, Basal cell carcinoma; KA, Keratoacanthoma; SCAP, Syringocystadenoma papilliferum; TB, Trichoblastoma.

^a^ Excluding warts.

^b^ Age and pathology of patients who were accompanied by multiple tumors.

BCC was the sole malignancy identified within our surgical pathology case series, with a total of 8 cases, of which 6 were adult cases, and 2 were pediatric cases (10.1 and 10.3 years). The mean age of patients with BCC was 37.3 years (range: 10.1–69 years, median: 38.5 years), significantly higher than that of those without (*p* < 0.001, Figure S3b). However, the average age of those with warts (15.4 years) was not significantly different than that of those without (12.5 years, *p* > 0.05, Figure S3e).

Multiple tumors appeared in patients aged 10–50 years, increasing with older age (Table 4), with the youngest a 10.3‐year‐old male with BCC and TB.

**FIGURE 5:**
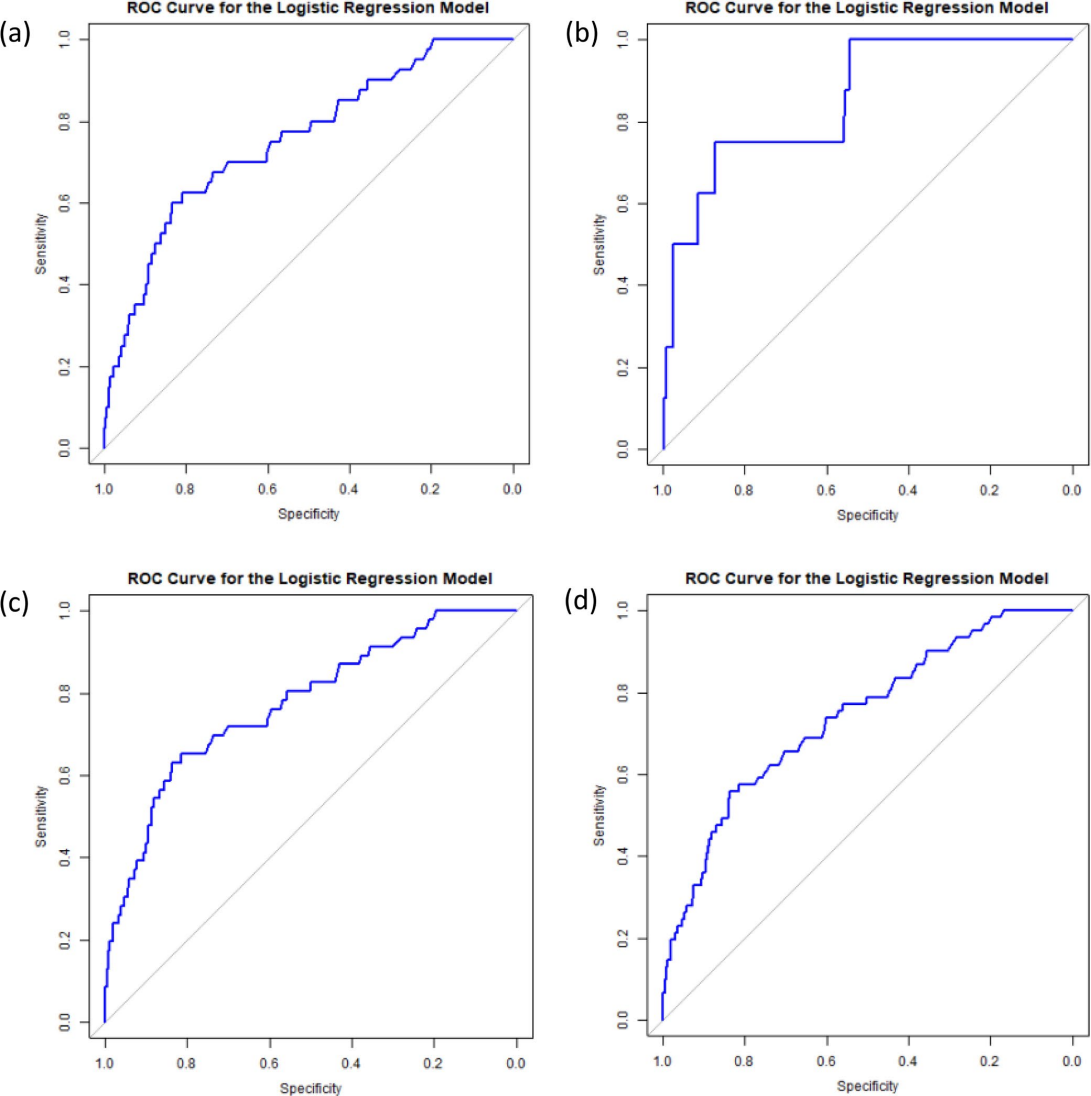
ROC curve for secondary tumors of NS. (a) ROC curve for benign tumors (excluding warts). (b) ROC curve for malignant tumor (BCC). (c) ROC curve for benign and malignant tumors (excluding warts). (d) ROC curve for benign and malignant tumors (including warts).

**FIGURE 6:**
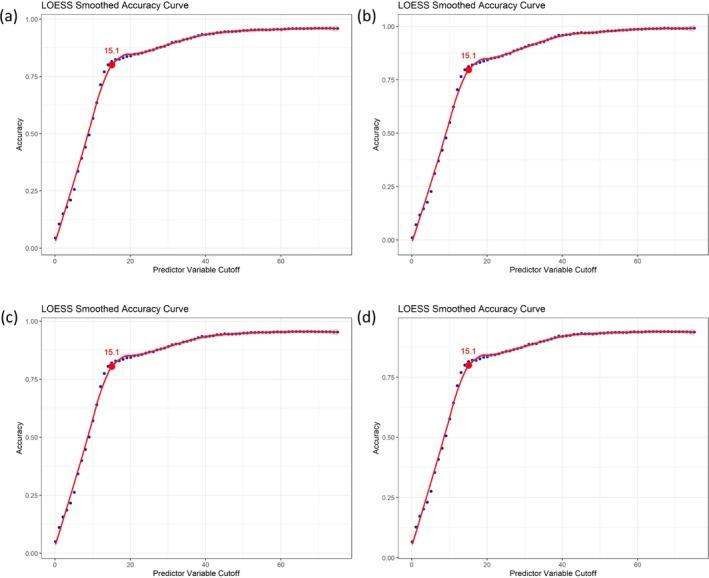
Cut‐off age for tumors of NS. (a) Cut‐off age for benign tumors (excluding warts). (b) Cut‐off age for malignant tumor. (c) Cut‐off age for tumors excluding warts. (d) Cut‐off age for tumors including warts.

Notably, we also identified that when NS lesions were resected after 15.1 years of age, there was a higher likelihood that secondary tumors were already present in the lesion (Figures 5 and 6).

### 3.3 Follow‐Up Survey

Among the 118 patients participating in the survey, most NS were identified at birth (61%) or shortly after (39%). Lesions showed scalp (79.7%) and face (12.7%) predominance. The proportion of lesions with rough texture (66.1% vs. 46.6%; *χ* ^2^ = 9.490, *p* < 0.05), and protruding profile (68.6% vs. 58.5%; *χ* ^2^ = 2.750, *p* > 0.05) seemed to increase at the time of initial surgery compared to at first discovery. At initial surgery, fourteen patients (11.9%) had a cerebriform appearance (Figure 2A), none later developed tumors, among whom only 3 underwent their first surgery as adults. Itching (34.7%), proliferative nodules (18.6%), and necrosis (12.7%) were commonly reported symptoms. In pediatric patients (< 12 years), pruritus (35.3%) and exudation (12.9%) proportions were high. Extracutaneous involvement such as ophthalmologic abnormalities (2 patients) or abnormal mental development (1 patient) occurred infrequently. Misdiagnoses like verrucous nevus (27.1%) and warts (6.8%) occurred before histological confirmation.

Previous CO_2_ laser treatment (3 cases) resulted in recurrence. After surgery, scar management included tension reduction (77 cases), scar gel/tape (38 cases), or fractional laser therapy (4 cases). Outcomes after surgery were favorable, with the average postoperative scar satisfaction score being 7.6, and the psychological burden scale decreasing from 4.4 to 2.6 (*p* < 0.05) for patients and from 5.6 to 3.2 (*p* < 0.05) for parents after surgery.

### 3.4 Literature Review

We identified 27 publications of large sample NS studies, and 19 were in English (Table S1). The largest included 997 patients.

Reported benign tumor incidence ranged from 2% to 39%, most commonly SCAP (1.1%–19.2%), trichilemmoma (1.1%–32.5%) and TB (1.6%–7.7%). Malignant tumor incidence ranged from 0.6% to 22.2%, most frequently BCC (0.6%–22.2%). However, some early high BCC rates may result from misdiagnosis of trichoblastomas [18]. Additional reported malignant histologies included Squamous Cell Carcinoma (SCC), Keratoacanthoma (KA), Sebaceous Epithelioma (SE), etc.

Treatment perspectives favored full‐thickness surgical excision over other methods like fulguration, electrocautery, or dermabrasion [5, 8, 9]. Opinions differed on timing: (1) some advocated for early childhood excision [8, 9] while others argued against this or supported monitoring instead of preemptive surgery [10, 11, 12, 13]. (2) Excision may only be needed for lesions with suspected tumor changes or aesthetic concerns [14, 15]. (3) All NS should have excision preemptively while allowing flexibility in timing [16]. (4) Excision was an option during childhood but strongly advised after puberty [6, 17].

In Asian studies, views ranged from early childhood excision [8] to elective surgery during childhood and strong recommendation for puberty surgery [17]. European studies proposed early childhood excision [9] to not supporting early intervention and favoring monitoring instead [10, 11, 13]. North American research suggested later childhood [5] or adolescent procedure [6] versus not requiring preemptive excision [12, 14]. Views were not discussed in some publications.

Data varied on ages of secondary malignant tumors. Some reports found no pediatric malignant tumors [11, 13], while others documented malignant tumors under 18 years [9, 16].

Representative case reports demonstrated multiple tumors can arise from NS, such as a case with 8 tumors and 2 cysts reported by Liu, Y. et al. [19], and a case with 3 malignant tumors reported by Hihara, M. et al. [20].

## 4 Discussion

This large retrospective study of 953 nevus sebaceous (NS) cases provides critical insights into clinical characteristics, management challenges, and the timing of intervention. Our findings not only corroborate prior observations but also reveal novel nuances, particularly regarding secondary tumor risks and resection timing.

### 4.1 Clinical and Histopathological Characteristics

Consistent with previous reports [5], NS lesions in our cohort predominantly involved the scalp and face, though with greater size variability (0.5–20 cm vs. 1–6 cm in prior studies). While diffuse and multifocal lesions tended to undergo earlier excision (mean age 6.7 vs. 13.3 years for facial lesions) in this single center, surgical timing did not significantly differ by anatomical location. The facial distribution pattern (cheeks > forehead > periauricular) in this series differed slightly from previous reports (forehead > cheeks > nose) [16], and the 32.5% misdiagnosis rate (most commonly as verrucous nevus and warts) underscores the importance of histopathological confirmation.

A subset of cases exhibited cerebriform sebaceous nevus (CNS), a rare variant first described in 1991 [21], despite its scarcity in literature [22, 23, 24, 25, 26, 27, 28, 29], 11.9% of our questionnaire participants presented with CNS. These cases showed minimal extracutaneous risk (only 1 case with central nervous system involvement) and no secondary tumors, likely due to early aesthetic‐driven excision [30].

### 4.2 Secondary Tumor Risks: Benign and Malignant

The life history of NS was divided into 3 phases [5]. In this series, the increasing trend in rates of epidermal proliferation and sebaceous gland hyperplasia was consistent with this theory.

Notably, this series represents one of the largest NS case series, with literatures reporting 10%–40% secondary tumor risk (2%–39% for benign tumors, typically SCAP, trichilemmoma, TB). Our 4.2% benign tumor rate aligns with these types, with variations likely due to differing age distributions across studies.

For malignant transformation, basal cell carcinoma (BCC) remains the most frequently reported malignancy. Earlier beliefs that half of NS cases developed BCC were later suggested to be inflated by misdiagnosed pilomatrixomas. Our 0.8% BCC rate aligns with subsequent reports (< 1%) [12, 13, 16]. Notably, while BCC was previously considered adult‐onset, our series identified two cases in 10‐year‐olds, echoing reports in cases as young as 15 and 9.7 years old [9, 16]. Other malignancies (e.g., SCC, KA, SE) were absent in our pediatric‐predominant cohort, reinforcing BCC as the primary concern.

### 4.3 Symptom Management and Treatment Strategies

Pediatric NS often presents with pruritus and exudation, symptoms frequently misinterpreted by parents as signs of tumor. However, these symptoms respond well to short‐term topical steroids like eczema (Figure 2B). Since 1965, complete excision has remained the gold standard over dermabrasion or laser therapies, as evidenced by high recurrence rates (e.g., CO_2_ laser) in our cohort. While younger children (< 5 years) frequently required general anesthesia, most older children tolerated procedures without it in our cohort.

### 4.4 Timing of Excision: Balancing Risks and Benefits

Debate remains around prophylactic NS excision timing (Table S1). Perspectives include advocating early childhood excision [8, 9] versus questioning necessity [10, 11, 12, 13] versus excising lesions with changes or cosmetic concerns [14, 15]. Most endorse elective childhood excision but recommend pubertal excision due to rising risk [6, 17]. North America literature seem to suggest relatively later intervention compared to that in Asia or Europe.

Our study observed increasing tumor incidence in resected NS lesions with age, with the earliest benign (4.7 years) and malignant (10.1 years) tumors in childhood. A critical finding was the 15.1‐year cutoff for tumor detection in the resected NS lesion, suggesting earlier‐than‐expected risk. Integrating these results with literature, we therefore propose our opinion on the timing of resection of NS.

We recommend that all patients with NS undergo close follow‐up starting at age 10, with surgical excision ideally performed before age 15. This recommendation is based on three key factors: the significant increase in secondary tumor risk after age 15, the substantial reduction in psychosocial distress for patients and parents following excision, and the ability of patients at this age to tolerate local anesthesia. However, management should be individualized, and earlier surgical intervention (including under general anesthesia when necessary) may be considered for lesions causing significant cosmetic or psychological impact, particularly those with diffuse distribution.

Our clinical data revealed cases of benign secondary tumors developing as early as 4.7 years of age. Therefore, in younger patients, the presence of any suspicious proliferative changes on clinical examination or dermoscopy should prompt consideration of biopsy or surgical excision.

Despite the overall low malignancy risk, lifelong monitoring is advised due to potential late‐onset tumors.

## 5 Limitations

This study's retrospective design inherently limits causal inferences. The recorded ages of secondary tumors reflect resection timing rather than true onset; thus, tumor development likely predates documented excision. Additionally, the predominance of pediatric cases may underestimate adult‐onset malignancies.

## 6 Conclusion

By synthesizing one of the largest NS cohorts to date, this work advances evidence‐based strategies for risk stratification and treatment timing. Future large‐scale prospective studies are needed to validate our proposed framework and refine personalized care paradigms.

## Ethics Statement

This study was approved by the Ethics Committee of Xinhua Hospital, Shanghai, China (No. XHEC‐D‐2021‐155). This research was performed in accordance with the ethical standards in the Declaration of Helsinki and its later amendments.

## Consent

Informed consent was obtained from all participants, and written consent forms were signed in accordance with institutional requirements.

## Conflicts of Interest

The authors declare no conflicts of interest.

## Supporting information


**Data S1:** jde17941‐sup‐0001‐DataS1.docx.

## Data Availability

The datasets analyzed during the current study are available from the corresponding author on reasonable request.
