# Supplementary material for: Should Be Removed or Taken Biopsy in Early Stage: A Retrospective Study of 953 Cases of Nevus Sebaceous From a Single Center
Source: J Dermatol. 2025 Sep 10;52(12):1763–74. doi: 10.1111/1346-8138.17941 (PMC12698902; doi:10.1111/1346-8138.17941)
Supplement: Supplementary file 1 — Data S1: jde17941‐sup‐0001‐DataS1.docx. [file JDE-52-1763-s001.docx]

**Supplemental materials**

**Title**：Should be removed or taken biopsies in early stage: A retrospective study of 953 cases of Nevus sebaceous

**Abbreviations**

NS: Nevus sebaceous;

CNS: Central Nervous System;

SCAP: Syringocystadenoma papilliferum;

BCC: Basel cell carcinoma;

KA: Keratoacanthoma;

SCC: Squamous Cell Carcinoma;

AE: Apocrine Epithelioma;

SE: Sebaceous Epithelioma;

TB: Trichoblastoma;

SpCC: Spindle cell squamous cell carcinoma;

IKA: Invasive Keratoacanthoma;

**Supplemental method**

***Literature review and data extraction***

We searched PubMed, web of science, and Ovid up to December 2023, using the key word ‘Nevus sebaceous’, and ‘Sebaceous nevus’. We identified 27 large sample studies (N>10), of which 19 were in English. Year of publication, language, author, patient population, area, number of lesions, percentage of benign tumors, type of benign tumors, percentage of malignant tumors, type of malignant tumors, and opinion on surgery were extracted.

**Epidermal naevus syndrome cases (ENS)**

In this series, isolated NS was seen in 99.5% of cases, 5 patients (0.5%) had multisystem involvement consistent with ENS. Lesions were diffusely distributed along Blaschko's lines in 4 cases. Two cases showed cerebriform appearing lesions. Neuroimaging found CNS involvement in 2 patients and 1 had mild electroencephalographic abnormalities. One presented with retinal vessel tortuosity. Genetic testing found 1 patient harbored a HRAS c.181C>A (p.Gln61Lys) gene mutation. Associated cutaneous lesions included epidermal nevi, complex nevi, and nevus giganteus.

**Supplemental tables**

Table S1. Literature review and summary of large sample case series studies of NS.

| **Year** | **Language** | **Author** | **Population** | **Area** | **No. of lesions** | **Benign tumors (%)** | **type of benign tumors (%)** | **Malignant tumors (%)** | **Type of malignant tumors** | **Opinion on surgery** |
| --- | --- | --- | --- | --- | --- | --- | --- | --- | --- | --- |
| 1965 | English | Mehregan and Pinkus | America | North America | 150 | 18 | SCAP (12%), Hidradenoma (4%), Infundibuloma (3.3%), Apocrine cystadenoma (2.6%) | 16 | 14% BCC, 2.6% KA, 2.6% SE | In many cases, it may be advisable to do preventive excision or plastic surgery later in childhood before the unsightly blossoming out in puberty that may be anticipated. Presence of apocrine glands requires full thickness excision. Superficial destruction of such lesions may actually provoke a papilliferous syringadenoma |
| 1970 | English | Jones and Heyl | America | North America | 140 | 39 | SCAP (19.2%), Basaloid proliferation (10%), 5% Others (syringomas,  apocrine cystadenomas,  osteomas) | 8.5 | 6.4% BCC, 0.7% SCC, 1.4% KA | There is only a small risk of a serious malignant tumor supervening in NS, and that simple surgical excision is usually adequate treatment. |
| 1979 | English | Domingo and Helwig | America | North America | 997 | 10 | N/A | 0.9 (BCCs were  excluded) | 0.4% Apocrine carcinoma, 0.3% Adnexal carcinoma with  pillar differentiation, 0.1% SCC, 0.1% SCC plus adnexal  carcinoma | Not mentioned. |
| 1985 | English | Morioka | Japan | Asia | 86 | 33 | Trichilemmoma (32.5%), SCAP (2.3%), 4.7% Others (verrucous  trichilemmal tumor,  proliferating  epidermoid cyst, syringoma,  apocrine nevus, apocrine  adenoma, sebaceous  adenoma) | 17.4 | 11.6% BCC (one had  associated SE and AE) | Not mentioned. |
| 1985 | English | Serpas and Hernandez | Salvador | North America | 29 | – | – | 6.8 | 6.8% BCC (one was  associated with leiomyoma) | NA |
| 1990 | English | Weng et al. | Taiwan | Asia | 62 | 11.4 | SCAP (11%), Trichilemmoma (6%) | 19.2 | 9.6% BCC, 9.6% SE | Early excision of the lesion in childhood before it enlarges and matures is recommended. Electrocautery, fulguration, or superficial destruction of the lesion is not recommended. Full-thickness, complete excision with primary reconstruction is the accepted treatment of choice. |
| 1995 | English | Chun et al. | America | North America | 165 | 5.4 | TB (3%), SCAP (1.8%) | 0 | 0 | Prophylactic excision of all nevus sebaceous is not warranted. Excision should be recommended only when benign or malignant neoplasms are clinically suspected or for cosmetic considerations. |
| 1999 | English | Beer et al. | Feldkirch, Austria and Zürich, Switzerland | Europe | 18 | 5.5 | Cystadenoma (in association  with BCC) | 22.2 | 22.2% BCC, One associated with KA | To continue surgical treatment of NS instead of dermabrasion or dermablation and to have the specimen examined histologically and, second, to excise such tumors as early in childhood as possible. |
| 2000 | English | Cribier et al. | France | Europe | 596 | 9.9 | SCAP (5%), TB (4.6%), Trichilemmoma (2.6%), Sebaceoma (2.1%), 1.3% Others (nevocellular  nevus, seborrheic keratosis) | 1.5 | 0.8% BCC, 0.7% KA | Because most tumors occurred in adults older than 40 years, our study suggests that prophylactic surgery in young children is of uncertain benefit. Clinical follow-up is probably sufficient, and even those cases with clinical changes often proved to be benign tumors or warts. |
| 2000 | English | Jaqueti et al. | Spanish | Europe | 155 | 36 | TB (7.7%), SCAP (6.4%), Sebomatricoma (5%)  16% Others (apocrine  hydrocystoma, apocrine  poroma, trichilemmoma,  desmoplastic trichilemmoma  primitive follicular induction,  ductal induction) | 0 | 0 | In our series, no examples of malignant neoplasms were identified. On the basis of these findings, the classically recommended treatment for this hamartoma, which consists of early excision to prevent the development of malignancy, seems to be inappropriate. |
| 2000 | English | Kaddu et al. | Austria | Europe | 316 | 7 | TB (7%) | 0.6 | 0.6% BCC | Most neoplasms developing in NS represent TB rather than BCC and that such neoplasms have characteristic histopathologic features that allow prompt recognition by conventional microscopy. |
| 2002 | English | Munoz-Perez et al. | Spain | Europe | 226 | 18.1 | SCAP (12.8%), TB (4.4%) | 3.5 | 3.5% BCC | The incidence of malignancy arising on SN was very low, indicating that prophylactic surgery of NS in children is not recommendable. Developmental defects should be investigated in order to evidence possible epidermal naevus syndrome associated with SN. |
| 2003 | English | Santibanez-Gallerani et al. | America | North America | 757 | 2 | Apocrine cystadenoma (0.8%) | 0 | 0 | No cases of basal cell cancer were found in the nevus sebaceous group. Recent studies in children corroborate these findings and question the need for prophylactic surgical removal of the nevus sebaceous. |
| 2008 | English | Simi et al. | India | Asia | 21 | 0 | 0 | 4.7 | 4.7% SCC | Though malignancy is uncommon, a cautious histologic analysis is mandated, especially if there are clinical changes in a lesion. |
| 2009 | English | Rosen et al. | America | Various ethnics | 631 | 2 | SCAP (1.1%), Melanocytic nevi (0.7%), 0.2% Others (apocrine cystadenoma, sebaceous trichoepithelioma, focal atypical syringoma) | 0.8 | 0.8% BCC | Malignant transformation of NS can occur in childhood or adolescence. We believe all NS should be excised; however, timing of excision can be flexible. Our data do not support age cutoffs or morphologic changes to determine optimal excision time. In conjunction with the treating physician, the parent and patient may weigh the small risk of malignant transformation of NS against the morbidity associated with excision and anesthesia. |
| 2010 | English | Manonukul and Kajornvuthidej | Thailand | Asia | 85 | 13.2 | Trichilemmoma (4.7%), TB (3.5%), SCAP (3.5%), Primitive follicular induction (8.2%), 3.5% Others (trichoepithelioma,  sebaceous adenoma, tumor  of follicular infundibulum) | 1.1 | 1.1% Mucoepidermoid carcinoma | The frequent occurrences of associated neoplasms in nevus sebaceous suggested that the prophylactic excision was the treatment of choice and should be considered. |
| 2014 | English | Idriss and Elston | America | North America | 707 | 18.9 | TB (7.4%), SCAP (5.2%), Apocrine/eccrine adenoma (2.1%), Trichilemmoma (1.1%), 3.1% Others (desmoplastic trichilemmoma, sebaceoma,  tumor of follicular infundibulum) | 2.5 | 1.1% BCC, 0.57% SCC including KA, 0.83% others (sebaceous carcinoma, apocrine carcinoma, microcystic adnexal carcinoma) | Most of the secondary neoplasms arising in association with nevus sebaceous are benign. As no malignant tumors were seen in children, we believe it is reasonable to delay surgical management until adolescence. |
| 2016 | English | Kambiz Kamyab-Hesari et al. | Iran | Asia | 168 | 5.9 | TB (2.3%), SCAP (1.2%), Trichilemmoma (1.7%) | 0 | 0 | Development of malignancies in NS is a rare phenomenon, and decision for excision of the lesion should be made after thorough evaluation of the pros and cons. |
| 2016 | English | Ming-Chun HSU et al. | Taiwan | Asia | 450 | 6.9 | SCAP (2.7%), TB (1.6%), trichilemmoma (1.6%), sebaceoma (0.7%), Apocrine adenoma (0.2%), Inverted follicular keratosis (0.2%) | 1.6 | 0.9% BCC, 0.2% SC, 0.2% SCC, 0.2% SpCC | Our study concludes that malignant transformation is rare in NS and occurs uniquely in adulthood. Prophylactic excision of NS can be elective during childhood but is strongly advocated at puberty due to the increased risk of malignant transformation with time. |
| 1969 | French | Michalowski | France | Europe | 160 | 28 | N/A | 21 | 21% BCC | Not mentioned. |
| 1986 | German | Smolin and Hundeiker | Germany | Europe | 181 | – | – | 12 | 11.6% BCC  0.5% PCC | All these secondary tumors had developed in postpubertal patients. |
| 1990 | Chinese | Chen et al. | Taiwan | Asia | 104 | 11.6 | SCAP (7.6%), Trichilemmoma (3.8%) | 8.5 | 5.7% BCC, 2.8% SE | Since a variety of tumors may develop in the NS after puberty, prophylactic removal of NS and close follow-up are suggested. |
| 1991 | Spanish | Perez Olivia et al. | Spanish | Europe | 40 | NA | NA | 12.5 | 12.5% BCC | NA |
| 1993 | French | Bonvalet et al. | Paris | Europe | 90 | 13.1 | SCAP (7% ),  6% Others (nodular  hidradenomas, chondroid  syringoma, trichilemmoma,  apocrine cystadenoma,  follicular poroma) | 12.1 | 12.1% BCC | NA |
| 1998 | Japanese | Minami and Kitano | Japan | Asia | 136 | 5.1 | Aprocrine cystadenoma (0.6%), SCAP (2.9%), TB (2.2%) | 7.3 | 2.9% BCC, 2.2% Sebaceous carcinoma, 2.2% SE | NA |
| 2004 | Arabic | Taklif and Jalilvand | NA | NA | 42 | 4.8 | Eccrine spiradenoma (2.4%), Trichilemmoma (2.4%) | 9.5 | 7.1% BCC, 2.4% IKA | NA |
| 2007 | Japanese | Ansai et al. | Japan | Asia | 243 | 33/243 | NA | 10/243 | NA | NA |

**Supplemental Figures**


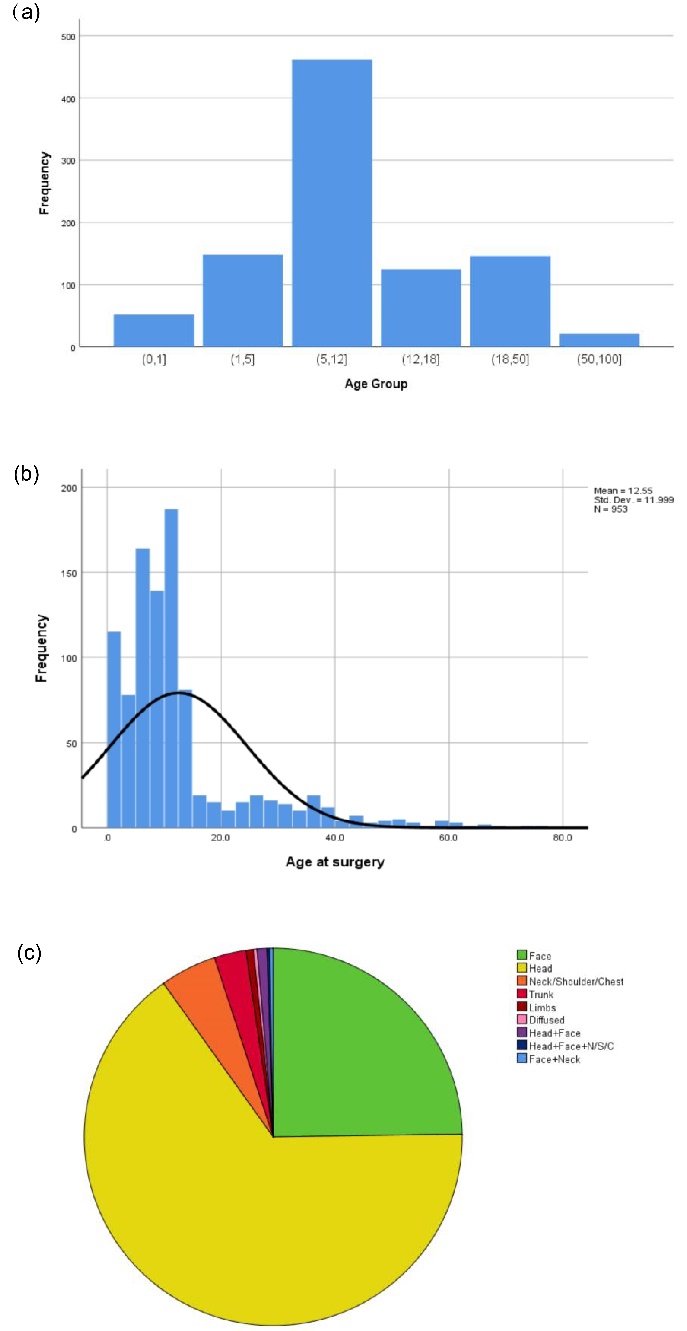


Figure S1. Age distribution and area distribution of NS surgery.

(a) A column chart of age segments at the time of surgery for patients with NS.

(b) A distribution graph of ages at the time of surgery for patients with NS.

(c) A pie chart of lesion locations for patients with NS.


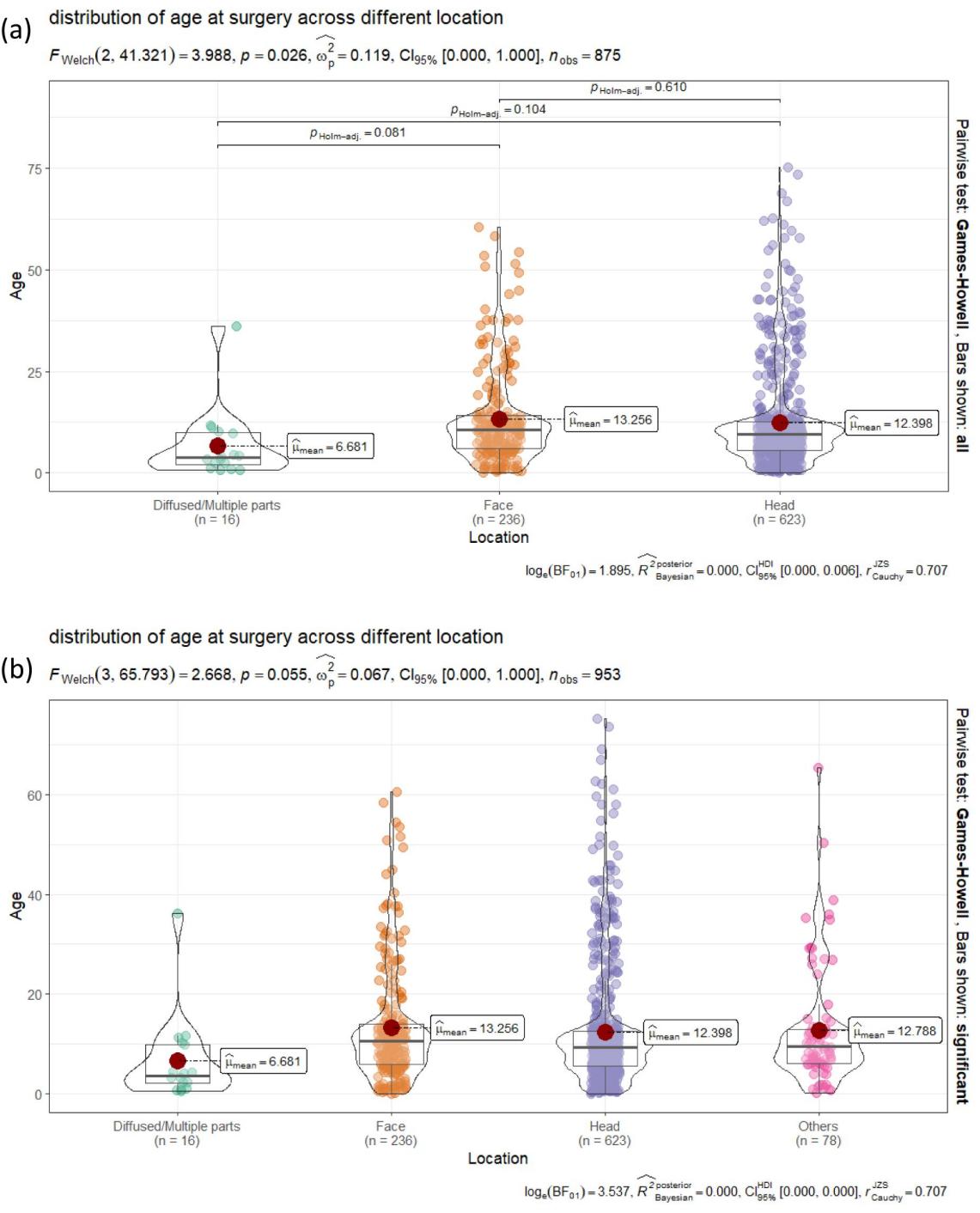


Figure S2. Comparison of age at surgery across different locations.

(a) There was an overall difference in the age at surgery for sebaceous gland nevi on the face, scalp and diffuse sites, but no significant differences were found between any two sites upon pairwise comparison.

(b) There were no significant overall differences in the age at surgery for sebaceous gland nevi on the face, scalp, diffuse sites and other sites.


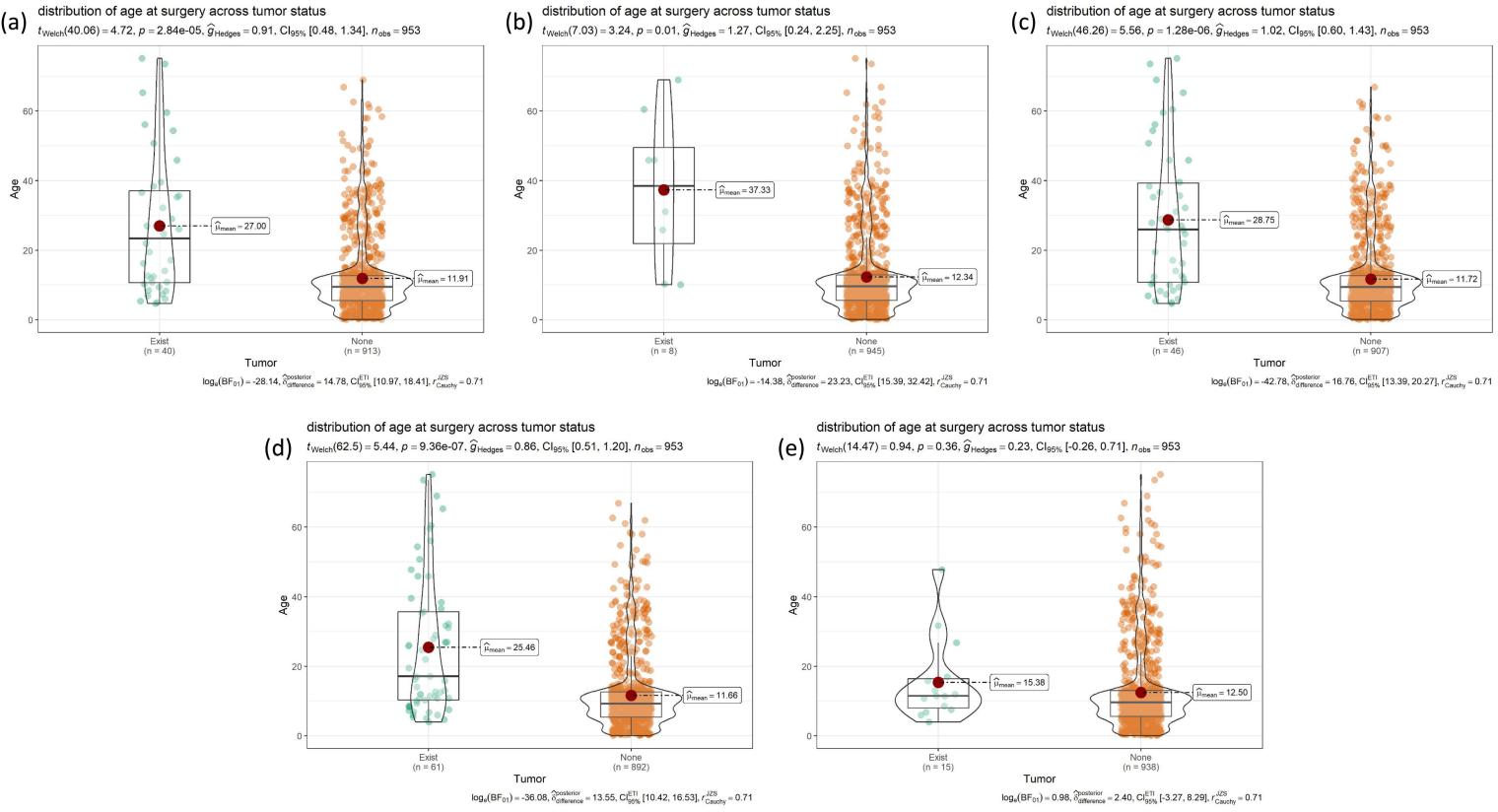


Figure S3. Comparison of age between patients with tumor vs without, patients with warts vs without.

1. Patients with benign tumors had a significantly older mean age (27.0 years) compared to patients without tumors (11.9 years), p <0.001.
2. Patients with malignant tumors had a significantly older mean age (37.3 years) compared to patients without malignant tumors (12.3 years), p <0.001.
3. Patients with tumors (excluding warts) had a significantly older mean age (28.8 years) compared to patients without tumors (11.7 years), p <0.001.
4. Patients with tumors (including warts) had a significantly older mean age (25.5 years) compared to patients without tumors (11.7 years), p <0.001.
5. No significant difference found between patients who have warts (15.4 years) and patients who have no warts (12.5 years), p>0.05.
